# Bacteriocins and Bacteriophages: Therapeutic Weapons for Gastrointestinal Diseases?

**DOI:** 10.3390/ijms20010183

**Published:** 2019-01-06

**Authors:** Loris Riccardo Lopetuso, Maria Ernestina Giorgio, Angela Saviano, Franco Scaldaferri, Antonio Gasbarrini, Giovanni Cammarota

**Affiliations:** 1 Istituto di Patologia Speciale Medica, Università Cattolica del Sacro Cuore, 00168 Roma, Italy; lopetusoloris@libero.it (L.R.L.); estin.gio3@gmail.com (M.E.G.); saviange@libero.it (A.S.); francoscaldaferri@gmail.com (F.S.); antonio.gasbarrini@unicatt.it (A.G.); 2 UOC Medicina Interna E Gastroenterologia, Area Gastroenterologia ed Oncologia Medica, Dipartimento di Scienze Gastroenterologiche, Endocrino-Metaboliche e Nefro-Urologiche, Fondazione Policlinico Universitario A. Gemelli IRCCS, 00168 Roma, Italy

**Keywords:** bacteriocins, bacteriophages, antibiotics, gastrointestinal diseases, dysbiosis, gut barrier, gut microbiota, virus

## Abstract

Bacteriocins are bactericidal peptides, ribosomally synthesized, with an inhibitory activity against diverse groups of undesirable microorganisms. Bacteriocins are produced by both gram-positive and gram-negative bacteria, and to a lesser extent by some archaea. Bacteriophages are viruses that are able to infect bacterial cells and force them to produce viral components, using a lytic or lysogenic cycle. They constitute a large community in the human gut called the phageome, the most abundant part of the gut virome. Bacteriocins and bacteriophages may have an influence on both human health and diseases, thanks to their ability to modulate the gut microbiota and regulate the competitive relationship among the different microorganisms, strains and cells living in the human intestine. In this review, we explore the role of bacteriocins and bacteriophages in the most frequent gastrointestinal diseases by dissecting their interaction with the complex environment of the human gut, analyzing a possible link with extra-intestinal diseases, and speculating on their possible therapeutic application with the end goal of promoting gut health.

## 1. Introduction

Bacteriocins are potent small and heat-stable bactericidal peptides [1] with antimicrobial properties against different groups of microorganisms [2].

They were first described in 1925, but the interest in their production, function and possible applications in medical areas has been growing only recently [2]. Diverse kinds of bacteriocins have been described by size, inhibitory mechanism, target cells, spectrum of action, interaction with the immune system and biochemical features [3]. Bacteriocins are produced by both gram-positive and gram-negative bacteria, and by some archaea [4]. They are synthetized by ribosomes and influenced by environmental factors (e.g., temperature, pH, and composition of the culture medium) [5,6].

Many bacteriocins are produced by the *Firmicutes* phylum, others belong to *Bacteroidetes* and the remaining percentage to the *Actinobacteria* and *Proteobacteria* phyla [7,8,9].

In particular, within the *Firmicutes* phylum, the major interest has involved the bacteriocins derived from the gram-positive, lactic acid bacteria (LAB), bacteria commonly used in food fermentation and well-represented both in the human gastrointestinal, respiratory and genital tract [10].

Lactic acid bacteria mainly produce two classes of bacteriocins [11]. Class I includes the modified-peptides known as “lanthionine-containing bacteriocins” (an example is nisin [12], and it can also include linaridin, azoline, cyanobactin, glycocin, sactiobiotic, thiopeptide, and lasso peptide) [3]; class II includes “non-lanthionine-containing bacteriocins” divided into four other subclasses (a,b,c,d) based on their size [12]. Subclass a (i.e., pediocin PA-1, sakacin A [13]) is active against *Lactobacillus*, *Enterococcus*, *Clostridium*, *Pediococcus* and *Leuconostoc* [14,15,16]. Subclass b (i.e., plantaricins E,F and J,K) [17] is made up of two antimicrobial-peptides that work only in combination (E with F and J with K) [18]. Subclass c (i.e., garvacin ML) is represented by circular bacteriocins [11], while subclass d (i.e., enterocin Q, L50) is composed of linear bacteriocins with an unknown role [11,19]. Class II also includes subclass II e (i.e., microcin E492).

Most of these bacteriocins are able to inhibit the pathogens’ growth, defend the producer and to play a role in “signaling” peptides [3]. Mechanistically, they can act as pore-forming agents, or membrane perturbers [20], or they can interfere with the cellular division processes. Moreover, bacteriocins possess anti-viral, spermicidal [2], and anti-cancer properties [21], and can enhance the positive effects of probiotic bacteria [22] (i.e., bacteriocins produced by the *Bifidobacterium* strain) [3].

Most of the above-mentioned functions are shared by bacteriophages. These are viruses that infect bacterial cells and force them to produce viral components, using diverse mechanisms such as the lytic or lysogenic cycle [23,24], but also to a lesser extent the pseudo-lysogenic cycle, the cryptic life cycle [25] or chronic infection [26,27]. Abortive infections rarely occur [28].

Bacteriophages constitute a large community of the human gut microbiota [29] and, together with viruses, are considered the most abundant organisms on the planet [30]. In the human colon they represent around 10^15^ cells [31]. Bacteriophages are classified according to their DNA or RNA, morphology (filamentous, polyhedral, pleomorphic, spiral, etc.), life cycle and their habitats [32,33]. They usually act on a narrow, closed and specific range of bacteria [34]. Broad-spectrum bacteriophages are rarely described in the literature [35]. In the human gut, the bacteriophages-family of Caudovirales (Siphoviridae, Myovirididae, Podoviridae) is the most abundant, followed by Microviridae [36]. In healthy individuals the balanced and inverse correlation between Caudovirales and Microviridae ensures the maintenance of a eubiotic state. In fact, bacteriophages regulate the bacterial population (shifting the ratio of both symbionts and pathobionts), control their metabolism [37] and mediate anti-inflammatory responses. They can interact with immune cells, inducing the production of pro-inflammatory cytokines, they can down-regulate the oxidative stress, reducing the reactive oxygen species, and they carry out both protective and immuno-modulating effects on gut-lymphoid tissue [38].

In this review, we explored the multiple relationships of bacteriocins and bacteriophages with the gut barrier and their role in the most common gastrointestinal diseases.

## 2. Relationship of Bacteriophages and Bacteriocins with the Gut Barrier 

In the gut mucosa, bacteriophages select specific bacteria by using horizontal gene transfer, influencing their rate of mutation and genetic variability, and thus modulating their abundance and diversity [39,40]. On the capsid they express Ig (Immunoglobulin)-like receptors, which interact with mucin glycoproteins and can regulate innate and acquired immunity [41]. Thus, bacteriophages can influence bacterial composition, modify their function and interaction with epithelial cells, and modulate the glycoproteic mucin layer and control other microorganism populations both directly and indirectly [38]. Moreover, they are dynamic entities that can translocate across the gut barrier and migrate into the peripheral blood and the peripheral tissue, activating the immune system [38]. They also have a complementary action on dendritic cells and can be considered both activators of inflammation and at the same time anti-inflammatory players [42]. The bacteriophages’ translocation across the gut barrier has been confirmed by different metagenomics studies that revealed their presence in ascitic, urine and blood samples [42,43]. In this scenario, their actions should not only be considered to be focused on the gastrointestinal tract, but also extended to other sites.

Further, bacteriocins act both on the immune system and on the inhibition of competitive strains by directly influencing the niche competition among commensals [44]. Bacteriocins are commonly used strategically by commensals to colonize and persist in the human gut. Their activities could resemble those of a “probiotic”. Indeed, they allow the survival of specific communities in the gastrointestinal tract by selecting strains that are able to resist modification by the host diet, the inhibition of natural defensins, bile salts and other killing factors, and colonization by other species, overall improving gut barrier function and the host immune response [45]. Studies on animal ilea have confirmed the potential effects of bacteriocin against pathogens, which led to positive changes in the gut microbiota composition. This is the case of Bacteriocin Abp118 [3], produced by *Lactobacillus salivarius UCC118* [13] or salivaricin P, produced by another *Lactobacillus salivarius* strain with a probiotic trait [3]. Interestingly, *Lactobacillus salivarius* expresses the srtA gene to tie it to the epithelial cell’s surface, before producing protective bacteriocins [3]. Bactofencin A or bacteriocin 21 produced by *Enterococcus faecalis* are able to kill multidrug resistant-bacteria and contribute to the regulation of the niche competition among intestinal bacteria [44]. Similarly, LAB bacteriocins exert their role against *Staphylococcus Aureus* [14], some vancomycin-resistant enterococci [44], *Salmonella enteritidis* [14], *Clostridium Difficile* [46] and *Listeria monocytogenes* [14]. More studies are needed to test the therapeutic potential of these findings. At the same time, it should be noted that not all changes observed in vitro have also been registered in vivo. This discrepancy is not surprising since several perturbing factors can deeply affect bacteriocin production and their activities.

## 3. Role of Bacteriophages in Gastrointestinal Chronic Inflammation

The role of bacteriocins and bacteriophages in most gastrointestinal diseases remains unknown [38]. Many studies have focused on the role they play in inflammatory bowel disease (IBD), such as Crohn’s disease (CD) and ulcerative colitis (UC) [31].

Recent analyses support the idea that the gut virome is involved in intestinal inflammation [47]. Bacteriophages can induce bacterial lysis leading to the release of nucleic acids, proteins and lipids, which are sources of pro-inflammatory stimuli and are able to provoke crucial dysbiotic alterations in CD and UC. An increased abundance of *Caudovirales* with a lower presence of *Coliphages* is found in IBD patients, both in CD and UC [38,48]. At the same time, bacteriophages can influence the abundance and diversity of bacteria in IBD with multifactorial and often unknown mechanisms. Usually, bacteriophages possess a narrow spectrum of action both on bacterial cells and on the immune system, which is activated by their coat proteins. They can act as antigens or ligands and can contribute to chronic intestinal inflammation [49]. This high diversity in phage-composition could represent a possible risk factor for developing IBD. On the other hand, bacteriophages could represent promising therapeutic tools to control inflammation. In this scenario, recent studies have demonstrated that bacteriophages are effective in reducing intestinal *E. coli* colonization [50,51], in particular the adherent invasive strain (AIEC) [52], which is considered to be involved in the maintenance of chronic inflammation in IBD [53]. Galtier et al. found three virulent bacteriophages that were able to replicate in ileal and colonic portions and feces from gut murine samples colonized with the prototype AIEC strain LF82. A single day of oral treatment with these bacteriophages significantly decreased the intestinal colonization of AIEC strain LF82. Furthermore, this single-dose reduced DSS-induced colitis symptoms over a 2-week period in mice colonized with LF82 [52].

In humans, a recent randomized trial of oral phage therapy in 120 children with acute bacterial diarrhea in Bangladesh did not report any adverse events, but failed to achieve intestinal amplification or improve the diarrhea outcome [54]. This could be due to low phage coverage and insufficient E. coli pathogen titers, which required higher phage doses. One should note that, since bacteriophages are part of the human commensal microbiota and because they are highly specific, they are likely to have a better safety profile than antibiotic therapy. Currently, a phase I double-blind randomized placebo-controlled trial is ongoing in quiescent CD to assess the safety of a lytic phage preparation containing seven bacteriophages targeting AIEC. This study involves participants with documented AIEC in the stool taking either a phage preparation or placebo for 2 weeks to evaluate adverse events or disease exacerbation. If safe and successful, the goal is to develop this as an adjuvant therapy in patients with CD and AIEC colonization.

Furthermore, the potential presence of bacteriophages in the blood represents a new, unexplored field. More studies are necessary to evaluate the opportunity of using bacteriophages for screening and predicting disease progression [31,55] and to understand the amount and types of bacterial species associated with a specific degree of disease. Similarly, the changes in the gut bacteriophage population, and the consequent modulation of commensal bacteria and the activation of pro-inflammatory strains in IBD require further analysis. Understanding these mechanisms could be useful to positively act on bacterial antibiotic-resistance or to calibrate the anti-inflammatory effect of specific bacteria [47,48].

## 4. Bacteriophages and Bacteriocins in Bacterial Food Infections

The antibacterial properties of bacteriophages and bacteriocins are exploited in food research. In particular, bacteriocins are used as food preservatives [12] both for dairy products [56] and for meat, fish [57], vegetables and fruits [58], being classified as partially purified bacteriocins, crude-fermented dairy bacteriocins and protective cultures bacteriocins [2].

Bacteriocins are considered natural and safe food additives after being ingested by the gastrointestinal tract [59]. They have the interesting properties of stability, antimicrobial effects, potency, and no flavor alteration [59].

There are different commercially available bacteriocins such as nisin (named Nisaplin) or Pediocin PA-1 against the growth of *Listeria monocytogenes* in meat products [60]. Other bacteriocins, such as those produced by Enterococci, seem to reduce the contamination of cheese due to animal feces [61], while Enterocin AS-48, Enterocin CCM4231 and EJ97 are used to protect both fermented and unfermented vegetables [60]. Bacteriocins can be added to food through the direct inoculation of the producer-strains as a concentrated fermented product [60] or as a gradual-release preparation. They have antimicrobial activity against gram-negative bacteria that infect foods, and this property could be empowered by combining bacteriocins with other compounds (e.g., organic acids, phenolic compounds). Other antimicrobial bacteriocins involved in food protection belong to the class I and II bacteriocins of Bacillus subtilis GAS101 and act against some gram-positive bacterial species such as *Staphylococcus Epidermidis* [62]. Bacteriocins are overall able to reduce the costs of food treatments and at the same time increase the product shelf-life.

Similarly, bacteriophages are used against *Salmonella*, *Campylobacter* and *Enterococcus* on food [28]. The efficacy of bacteriophages is reduced by their spectrum of action, which is oriented against specific serotypes, species or strains of bacteria [28]. However, their narrow spectrum could be more advantageous than antibiotics and may have a lower impact on the other components of the gut microbiota [33].

Bacteriophages can also control the production of some pathogenic toxins such as Cholera, Shiga and Pertussis, and interfere with mechanisms of antibiotic resistance [48]. In the past, they have been used to control the epidemic of bloody diarrhea in Germany caused by *E. coli* strain 0104:H4 through the production of Shiga toxins [63].

Finally, an alteration of gut virome composition could also be found in recurrent *Clostridium Difficile* infection [64]. Interestingly, the presence of a complex of bacteriophages in fecal mass used for therapeutic fecal microbiota transplantation (FMT) has been demonstrated to improve clinical outcomes of this treatment [65].

## 5. Role of Bacteriophages and Bacteriocins in Extra-Intestinal Diseases

Bacteriophage therapy has strong potential as a treatment for many extra-intestinal diseases, and in particular in those correlated to bacterial infections [66]. Indeed, in chronic bacterial rhino-sinusitis, after twenty days of topical application, a bacteriophage cocktail (P68, K710) is able to control a broad range of *Staphylococcus Aureus* (*S. aureus*) strains, including the methicillin-resistant strain (MRSA) [66]. A common manifestation of invasive *S. aureus* infection is osteomyelitis. *S. aureus* triggers many profound alterations in bone remodeling. Bacteriophage therapy has been applied with promising results, but more data are needed to confirm these findings [67].

In animal models, it has been reported that bacteriophages are able to control *Pseudomonas* lung infections [46]. In fact, two bacteriophages, φMR299-2 and φNH-4, have been proven to induce the formation of a biofilm on lung cells useful in controlling these infections. Hypothetically, they could have beneficial effects in patients with cystic fibrosis who are mostly exposed to this microorganism [68].

Bacteriophage therapy has been also used successfully for acne treatment. Acne has a multifactorial etiology and the inflammatory follicular response caused by the gram-positive skin bacterium *Propionibacterium Acnes* seems to play a primary role. The most common first line treatment is based on topical antimicrobial agents or oral antibiotics. Recent data suggest an alternative treatment, both for acne and for others bacterial skin infections, based on the use of a lytic bacteriophage preparation able to kill specific bacterial cells [69].

Further advantages of bacteriophage treatment have been demonstrated for diabetic foot ulcer healing. A commercial topical preparation of staphylococcal bacteriophage Sb-1 is effective when antibiotic treatment is unsuccessful [70].

On the contrary, bacteriocins such as pyocin were unable to control *Pseudomonas* lung infections in patients with cystic fibrosis. In fact, despite initial positive laboratory experiments, subsequent studies have underlined no evidence of beneficial effects for this bacteriocin [71].

Another frequent infection is provoked by *Streptococcus Pneumoniae*, which is responsible for pneumonia, bacteremia and meningitis, in particular in children. Pneumococcal disease benefit from vaccine and antibiotic treatment, but resistance is increasing. *S. Pneumoniae* is common in the nasopharynx of children, and it produces a circular bacteriocin, known as pneumocyclicin, to attack other bacteria and to protect itself against the immune system. This bacteriocin is similar to the other circular bacteriocins produced by gram-positive bacteria, and it could represent an important target thanks to its correlation to antibiotic resistance mechanisms [72].

Overall, bacteriophage therapy could be very beneficial thanks to its action against all types of pathogens, including those that are multi-drug resistant. A positive aspect of their narrow spectrum is the possibility of preserving the existing microbiome. Bacteriophages also have lower side effects, a wide distribution after their administration, and a possible inhibitory effect on the inflammatory response. They are cost effective and some studies underline their efficacy in comparison with antibiotics [33]. Further, bacteriocins could be very useful in fighting pathogens. They are easier to modify through bioengineering and have targeted activity against specific microorganisms. Numerous studies are trying to prove their use as a natural defense and as an alternative to antibiotics [12] in peculiar cases such as pregnant women and in individuals with contraindications to antibiotic use [73].

## 6. Bacteriocins, Bacteriophages and Cancer

Bacteriocins and bacteriophages are gaining great importance in the medical oncology field. Both are able to induce an immune modulatory response against T and B cells [74], which are involved in the control of cancerous pathways [75]. In addition, they can stimulate cytokine secretion [76] and modify the tumor microenvironment to make anticancer treatment more effective.

Bacteriophages display a huge genetic flexibility and consequently a variety of surface modifications that can be used as a basis for phage display methodology. These manipulations could potentially lead to the targeted delivery of therapeutic genes. Furthermore, their strong safety profile allows their potential application as cancer gene therapy platforms. The combination of phage display with combinatorial technology has produced the organization of phage libraries, transforming phage display into a high throughput technology. Indeed, random peptide libraries are one of the most important phage libraries, as they offer a huge source of clinically useful peptide ligands [77]. Peptides represent promising pharmaceutical tools in the oncologic field with significant advantages, including the low costs of synthesis, efficient membrane penetration and the absence of immunogenicity. Phage peptide libraries can be interrogated against several oncologic targets such as cancer-homing ligands, and they serve as gene therapy vectors towards malignant cells [77]. Thanks to this method, a large number of peptide ligands can be produced through the addition of specific genetic fragments into genes encoding phage capsid proteins [76]. In this scenario, a filamentous phage is used to vector a functionally active green fluorescent protein into mammalian cells by exerting a mechanism commonly used by fibroblast growth factor for cell internalization [78]. Another group was able to inhibit vascular endothelial growth factor activity and thus angiogenesis through the use of a phage display library [79]. Moreover, these peptides can diminish tumor metastasis and block specific enzymes necessary for tumor progression [76]. In fact, the affinity of the phage T4 (wt4) and its substrain HAP1 with melanoma cells was used efficiently to inhibit lung metastasis in mice [80].

Bacteriocins share similar anticancer properties. In particular, Colicin A and Colicin E1 revealed inhibitory activity against the growth of eleven different tumor-cell lines [81]. Similarly, colicin D and colicin E2 showed an inhibitory effect against murine leukemia cells P388 and colicin E3 suppressed the malignant transformation of a chicken monoblast line [59]. Further, other colicins produced by *E. coli* strains were able to act against human colorectal carcinoma cells [59].

Finally, in mice, nisin was effective in controlling head and neck squamous cell carcinoma and oral cancer. The effects of this treatment resulted in the reduction of tumor volumes and was correlated with an increased cellular apoptosis mediated by CHAC1 expression, a cation transport regulator and apoptosis mediator [82].

## 7. Conclusions

In summary, bacteriophages and bacteriocins share significant potentially beneficial effects on human health (Table 1). In particular, bacteriocins could fill a gap in medical and food industry applications by playing a role as a “natural” and “safe” antimicrobial agent in the near future. They can regulate competitive interactions in the microbial community. Their narrow-target activity, surprising specificity, high stability and low toxicity make them an alternative or complement to current antibiotics. They could play a key role in antibiotic resistance and could become a useful approach in the treatment of infectious diseases. Moreover, thanks to their non-immunogenicity and ability to modulate cancer cell proliferation, they could act as potential synergistic agents with current conventional cancer treatments. Likewise, bacteriophages could share similar properties of effectiveness and safety in various medical fields (e.g., the modulation of chronic inflammation, antibiotic and cancer therapy, food safety). For this reason, in the world of nanotechnology and nanomaterials, they are emerging as valuable rising stars for modulating the gut barrier and restoring the overall homeostasis of the gut community.

## Figures and Tables

**Table 1:** An overview of bacteriocins, bacteriophages and antibiotics properties.

	Bacteriocins	Bacteriophages	Antibiotics
**Classes**	Class I (lanthionine-containing bacteriocins) Class II (non-lanthionine-containing bacteriocins): - IIa - IIb - IIc - IId - IIe Class III Class IV* (also containing lipid or carbohydrate and not only proteins)	Four classes based on the genetic composition: dsDNA ssRNA dsRNA ssDNA	β-Lactams Aminoglycosides Chloramphenicol Glycopeptides Ansamycins Streptogramins Sulfonamides Tetracyclines Macrolides Oxazolidinones Quinolones Lipopeptides
**Inhibitory mechanism** **(mechanisms of action)**	-Inhibit the pathogens’ growth, acting as pore-forming agents, membrane perturbers. -Dissipate the transmembrane electrical potential, leading to cell death. -Interfere with cellular division processes.	-Infect and use bacterial cells resources through: lytic cycle lysogenic cycle or pseudo-lysogenic cycle cryptic life cycle chronic infection -Inhibitory activity against pathogens’ growth through their combined action on both gut microflora species and immune system cells. -Interfere with bacterial cellular replication and transcriptional processes.	-Inhibit the biosynthesis of bacteria cell walls. -Inhibit the synthesis of proteins. -Inhibit the synthesis of RNA. -Interfere with bacterial DNA replication and transcription. -Disrupt multiple bacteria cell membrane functions.
**Target cells** **(spectrum of action)**	Mainly narrow spectrum on: -Bacterial cells-Viral cells -B and T Lymphocytes	Mainly narrow spectrum on: -Bacterial cells -Archaea	Narrow or broad spectrum on: -Bacterial cells -Parasites
**Size**	Small or large peptides (from less than 5kDa to 90 kDa)	Short or Long (from 24 to 200 nm)	
**Morphology (shape)**	Linear Globular Circular	Filamentous Icosahedral, polyhedral Pleomorphic Spiral Isometric With or without tails (contractile or non-contractile) With or without an envelope With or without a capsid	Heterogeneous
**Administration**	Mainly oral; intravenous, intranasal, intraperitoneal, subcutaneous (studies on animal models)	Intramuscular, intravenous, topical (studies on animal models)	Oral, intramuscular, intravenous, topical
**Application**	Food preservatives, treatment of intestinal and extraintestinal infections	Models for studying viral transformation, vehicles for vaccine delivery, synthesis of novel polypeptides, control of environmental and dangerous bacterial cell growth	Treatment or prevention of bacterial infections and in specific cases of protozoan infections.
**Side effects**	More studies are needed to test this		Allergic reactions, hypersensitivity, diarrhea, fever, nausea are the most common.
**Resistance**	Potential application to fight antibiotic resistance and act against the current multi-drug resistant pathogens		Very common

## Abbreviations

IBD	Inflammatory Bowel Diseases
CD	Crohn’s Disease
UC	Ulcerative Colitis
MRSA	Methicillin-Resistant *S. aureus*
